# Genome sequencing for rightward hemispheric language dominance

**DOI:** 10.1111/gbb.12572

**Published:** 2019-04-23

**Authors:** Amaia Carrion‐Castillo, Lise Van der Haegen, Nathalie Tzourio‐Mazoyer, Tulya Kavaklioglu, Solveig Badillo, Marie Chavent, Jérôme Saracco, Marc Brysbaert, Simon E. Fisher, Bernard Mazoyer, Clyde Francks

**Affiliations:** ^1^ Language and Genetics Department Max Planck Institute for Psycholinguistics Nijmegen The Netherlands; ^2^ Department of Experimental Psychology Ghent Institute for Functional and Metabolic Imaging, Ghent University Ghent Belgium; ^3^ Groupe d'Imagerie Neurofonctionnelle, Institut des Maladies Neurodégénératives, Centre National de la Recherche Scientifique, Commissariat à l'Energie Atomique et Université de Bordeaux Bordeaux France; ^4^ Institut de Mathématiques de Bordeaux, Centre National de la Recherche Scientifique, Institut National de la Recherche en Informatique et Automatique et Université de Bordeaux Bordeaux France; ^5^ Donders Institute for Brain, Cognition and Behaviour Radboud University Nijmegen The Netherlands

**Keywords:** brain asymmetry, DNA, dominance, functional MRI, genetics, hemispheric lateralization, language, laterality, next generation sequencing, whole genome sequencing

## Abstract

Most people have left‐hemisphere dominance for various aspects of language processing, but only roughly 1% of the adult population has atypically reversed, rightward hemispheric language dominance (RHLD). The genetic‐developmental program that underlies leftward language laterality is unknown, as are the causes of atypical variation. We performed an exploratory whole‐genome‐sequencing study, with the hypothesis that strongly penetrant, rare genetic mutations might sometimes be involved in RHLD. This was by analogy with situs inversus of the visceral organs (left‐right mirror reversal of the heart, lungs and so on), which is sometimes due to monogenic mutations. The genomes of 33 subjects with RHLD were sequenced and analyzed with reference to large population‐genetic data sets, as well as 34 subjects (14 left‐handed) with typical language laterality. The sample was powered to detect rare, highly penetrant, monogenic effects if they would be present in at least 10 of the 33 RHLD cases and no controls, but no individual genes had mutations in more than five RHLD cases while being un‐mutated in controls. A hypothesis derived from invertebrate mechanisms of left‐right axis formation led to the detection of an increased mutation load, in RHLD subjects, within genes involved with the actin cytoskeleton. The latter finding offers a first, tentative insight into molecular genetic influences on hemispheric language dominance.

## Graphical abstract

Study design of the genome sequencing for rightward hemispheric language dominance. Images are shown from an example subject with typical left‐hemisphere language dominance, and an example subject with atypical rightward hemispheric language dominance, as assessed by functional magnetic resonance imaging. Genomic analysis was focused on rare, protein‐altering variants within genes and candidate gene‐sets.

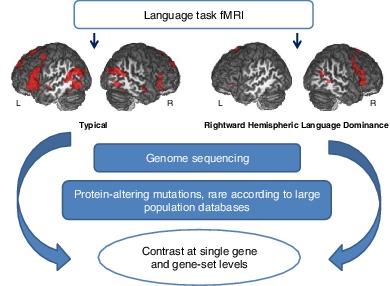

## 1 INTRODUCTION

Noninvasive imaging methods such as functional magnetic resonance imaging (fMRI) have shown that roughly 85% of people have left‐hemisphere language dominance, while most remaining people are ambilateral for language, and only a small minority of around 1% show rightward hemisphere language dominance (RHLD).^1^, ^2^, ^3^ The degree of laterality assessed with fMRI varies with the type of language task used and is usually more pronounced for language production than perception tasks.^4^ Roughly 90% of people are right‐handed, 10% left‐handed and a small remainder ambidextrous.^5^ Although more than 70% of left‐handers have left‐hemisphere language dominance,^3^ over 90% of people with RHLD are also left‐handed.^3^ Therefore, RHLD usually involves a broader re‐organization of left‐right laterality than purely for language functions, but may represent an etiological group that is distinct from the bulk of left‐handers.

Gene expression and in utero ultrasound studies of human embryos have indicated that lateralized development is already underway in the human central nervous system by 5 to 8 weeks post‐conception,^6^, ^7^, ^8^ which indicates a genetic‐developmental program underlying the typical form of functional brain laterality. One study reported a nonsignificant heritability (<1%) for the laterality of speech sound perception, based on the dichotic listening method, and considering the full range of trait variation from left‐ to right‐ear‐advantage.^9^ However, atypical functional language dominance, that is, a categorical trait defined to include both RHLD and ambilateral dominance, has been shown to have a heritability of roughly 30%, measured with functional transcranial Doppler sonography during language production.^9^, ^10^ There have been no twin or family‐based studies of RHLD heritability itself, likely due to the rarity of the trait. Twin and family studies have reported moderate heritability estimates for left‐handedness (24%‐39%),^10^, ^11^ although heritability estimates based on genomic similarity between unrelated people in the general population are much lower for left‐handedness (heritability = 1%‐3%).^12^, ^13^

Regardless, molecular mechanisms for the initial “symmetry breaking” process in the mammalian brain, that is, for establishing a left‐right axis in the very early embryo, remain unknown.^14^ In contrast, much is known about the developmental origins of asymmetry of the visceral organs (ie, heart, lungs and so on). Increased activation of the nodal signaling cascade on the left side of an early embryonic structure, called the node, ultimately results in asymmetric organogenesis.^15^ Motile cilia within the node are important for this process, because their unidirectional rotation, arising from the chirality of their protein constituents, produces a right‐to‐left fluid flow that triggers left‐sided nodal expression.^15^, ^16^ Monogenic mutations in genes that encode components of motile cilia, or otherwise affect ciliary functions, can cause the disorder primary ciliary dyskinesia (PCD) together with situs inversus totalis (SIT)*,* a condition affecting roughly 1/6000 to 1/8000 people, in which the visceral organs are placed as the mirror image of the usual arrangement.^16^, ^17^ PCD with SIT is a genetically heterogeneous condition, which can be caused by mutations in at least 37 different genes,^18^ although one gene accounts for 15% to 28% of cases (*DNAH5*).^19^, ^20^

Intriguingly, people with PCD and SIT do not show an increased rate of RHLD or left‐handedness, which suggests a fundamental dissociation between nodal‐ciliary mechanisms of visceral axis formation and the brain functional lateralities for language and hand dominance.^21^, ^22^, ^23^ Thus, the typical form of human brain functional laterality may instead originate from a genetic‐developmental mechanism that is brain‐intrinsic. Recent studies in *Drosophila* have showed that cellular chirality induces left‐right asymmetry of individual organs in an organ‐intrinsic manner, without being induced by the ciliary‐nodal pathway.^24^, ^25^, ^26^, ^27^ In these mechanisms, chirality is a transient property of whole cell morphology at key points in embryonic development.^24^ A role of actin‐related genes in establishing cellular chirality has been observed in both invertebrate (*Drosophila,* snail)^24^, ^25^, ^26^, ^27^ and vertebrate models (cultured cells, frog, zebrafish),^24^, ^28^, ^29^ suggesting that this mechanism is important to establish left–right organ asymmetry across bilaterian groups. Apart from the cilia‐related nodal signaling pathway, cellular chirality is the only biological mechanism that has been shown to give rise to organ asymmetry in multicellular animals, of which we are aware.

**Figure 1:**
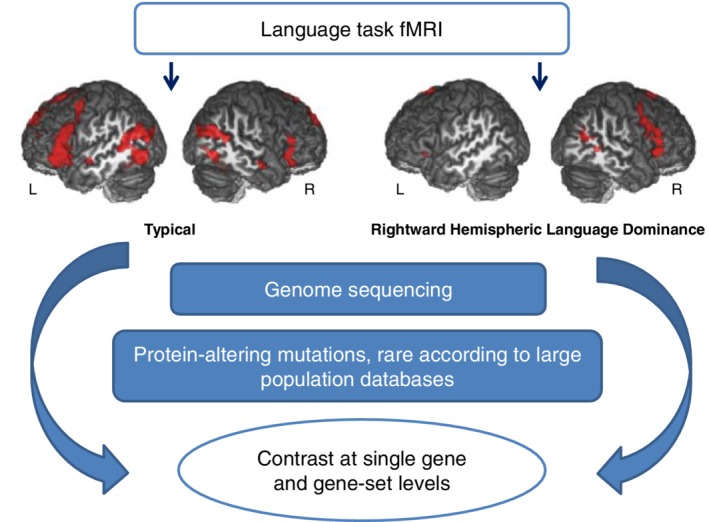
Schematic figure showing the study design. Images are shown from an example subject with typical left‐hemisphere language dominance, and an example subject with atypical RHLD, as assessed by fMRI. Genomic analysis was focused on rare, protein‐altering variants within genes and candidate gene‐sets

Recent analyses using the UK biobank data set, based on more than 300 000 participants, have reported that alleles of the microtubule‐associated gene *MAP2* have very small effects on the probability of becoming left‐handed, as well as some other loci which did not clearly implicate individual genes.^30^, ^31^ However, the rarer trait of RHLD, found in only roughly 10% of left‐handers and less than 1% of right‐handers, has not been subject to any previous molecular genetic studies. By analogy with SIT, here we investigated whether RHLD might sometimes arise due to high‐penetrance genetic mutations. We sequenced the genomes of 33 people with RHLD as assessed using fMRI, as well as 34 typically lateralized subjects (20 right‐handed, 14 left‐handed) and interrogated the data with reference to large population genetic databases (Figure 1).

As this was an exploratory study, we performed separate analyses under recessive and dominant models, allowing for allelic heterogeneity (different causative mutations within a given gene) or genetic heterogeneity (causative mutations in different genes). We also tested for an increased rate of rare mutations in RHLD within specific candidate gene sets, in case an increased load of mutations affecting specific biological processes might increase the chance of having RHLD. The candidate sets included genes involved in visceral laterality or the actin cytoskeleton, as well as a set of 18 genes which have been tentatively associated with human brain laterality in previous studies.^14^, ^30^, ^32^

## 2 METHODS

### 2.1 Data sets and functional laterality measurement

**Figure 2:**
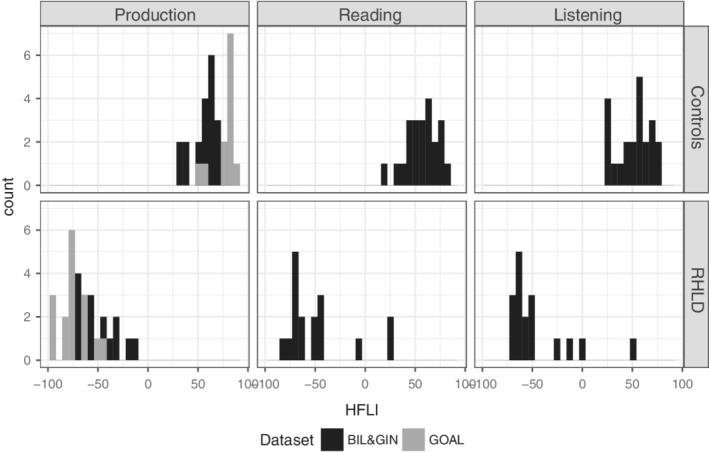
HFLI distributions for the language task contrasts within RHLD and control subjects. Negative HFLIs indicate rightward functional laterality. Note that GOAL samples were only assessed using Production HFLI

A total of 67 participants (33 with RHLD) were included in the present study, all of whom gave written informed consent. All RHLD subjects except one were left‐handed (Edinburgh Handedness Inventory [EHI] median = −87.50), while the controls included 14 left‐handed and 20 right‐handed participants (EHI median = 76.39; this composition allowed us to perform post hoc analysis using control groups of different handedness, see below). Summary statistics for language laterality measures and handedness are provided in Table 1, Figure 2 and Figure S2.

**Table 1:** Summary statistics for language laterality measures and handedness, within the 67 participants of this study

Data set	Group	N	Sex (M/F)	Handedness (LH/RH)	EHI	HFLIPROD	HFLIREAD	HFLILIST
BIL&GIN	RHLD	17	8/9	16/1	−22.92 [−100;100]	−58 [−72;‐15]	−61 [−84;24]	−59 [−72;52]
	Controls	22	10/12	14/8	−77.78 [−100;100]	61 [29;83]	59 [16;84]	57 [25;79]
GOAL	RHLD	16	4/12	16/0	−100 [−100;‐16]	−77 [−94;‐45]	‐	‐
	Controls	12	0/12	0/12	90.5 [67; 100]	83 [49;90]	‐	‐

*Note*: See also Figure 2.

Abbreviations: EHI, Edinburgh Handedness Inventory score: median [min‐max]. Median [min; max] values are shown for the three HFLI indexes. PROD, production; READ, reading; LIST, listening.

The subjects in this study were recruited from two separate sources, that is, the BIL&GIN data set (France) and the GOAL data set (Belgium).

### 2.2 BIL&GIN

Seventeen RHLD subjects and 22 controls were drawn from a larger data set of healthy, young adults, balanced for handedness (N = 297, of which 153 left‐handers).^3^ Informed consent was obtained from all participants, and the study was approved by the Basse‐Normandie local ethics committee (reference: CPP‐2006‐16).

We studied hemispheric lateralization for three language tasks, namely production, reading and listening, using fMRI to calculate Global Hemispheric Functional Laterality Indexes (HFLIs), as described previously.^33^ Each participant underwent a slow event‐related functional MRI protocol including three runs, one for each language task, presented in a random order. The three runs followed the same structure, alternating execution of the task at the sentence level and at the word list level. Word lists used in the tasks consisted of ordered lists of the months of the year or days of the week. fMRI was performed on a Philips Achieva 3Tesla MRI scanner. For each run, functional volumes were acquired with a T_2_^*^‐weighted echo planar imaging acquisition (192 volumes; repetition time (TR) = 2 seconds; echo time (TE) = 35 ms; flip angle = 80°; 31 axial slices; 3.75mm^3^ isotropic voxel size).

fMRI data analysis was performed using the SPM5 software (http://www.fil.ion.ucl.ac.uk/spm/). Scans of each participant and each run were normalized to our site‐specific template, corrected for motion during the run, and then warped into the standard montreal neurological institute (MNI) space using a tri‐linear interpolation, with subsequent smoothing using a 6‐mm full width at half maximum (FWHM) Gaussian filtering. We then computed for each participant the BOLD signal difference maps and associated t‐maps corresponding to the “sentence vs word‐list” contrast for the production, reading and listening runs. For each individual and each language task, we computed a HFLI using the LI‐toolbox applied to the individual contrast t‐map of the considered language task.^34^

A two‐step procedure was then implemented to select RHLD subjects and typically lateralized controls. We first selected the 10 individuals previously identified as strongly right‐lateralized in this data set using a stringent criterion based only on language production (HFLI for language production < −50).^3^ Then, to identify individuals exhibiting a right‐lateralized profile in all three language conditions, but who may have been overlooked in the first‐step, we modeled the joint distribution of the three HFLI using a mixture of 3D Gaussian functions and applied a robust consensus clustering approach.^35^ This second step uncovered 14 individuals having HFLI values < −15 for each of the three language conditions, including seven of those already selected in the first step. In total, 17 subjects were thus identified as having RHLD on the basis of their HFLIs for production, reading and listening. These 17, plus another 22 control subjects with typical left‐hemisphere language dominance, comprised the 39 BIL&GIN participants of the present study. HFLI distributions for RHLD and controls subjects are shown in Figure 2. The median age of RHLD subjects was 23 years, range 19 to 38 years, and for controls the median age was also 23 years, range 19 to 38 years. Information on sex is given in Table 1. We deliberately over‐represented left‐handedness in our selection of control subjects (14 left‐handed out of 22) to carry out post hoc analysis with respect to handedness (see Results). Handedness was assessed based on the Edinburgh inventory.^36^

## 3 GOAL

Sixteen RHLD participants were selected from a larger data set of healthy left‐handers (N = 250)^37^ that was first evaluated using the behavioral visual half field task to identify likely RHLD subjects, and then confirmed using fMRI to calculate Global HFLIs based on a language production task.^2^ Participants were asked to covertly think of as many words as possible beginning with a letter presented in the middle of the screen for 15 seconds. Ten different letters were presented in randomized order. The baseline condition consisted of 10 15‐second blocks with silent repetition of the non‐word baba. Experimental and baseline blocks were alternated with 20 rest periods of again 15 seconds, during which a horizontal line was displayed at the screen center. Images were acquired on a 3‐Tesla Siemens Trio MRI scanner (Siemens Medical Systems, Erlangen, Germany) with an 8‐channel radiofrequency head coil. First, a high‐resolution anatomical image was collected using a T1‐weighted 3D MPRAGE sequence (TR = 1550 ms, TE = 2.39 ms, image matrix = 256 × 256, FOV = 220 mm, flip angle = 9°, voxel size = 0.9 mm × 0.9 mm × 0.9 mm). Functional images were then obtained using a T_2_^*^‐weighted gradient‐echo EPI sequence. Forty axial slices covering the whole brain were acquired (TR = 2630 ms; TE = 35 ms; flip angle = 80^°^; image matrix = 64 × 64, FOV = 224 mm, slice thickness = 3.0 cm, distance factor = 17%, and voxel size = 3.5 mm × 3.5 mm × 3 mm).

The 16 strongly right‐lateralized individuals all met a stringent criterion for RHLD (HFLI for language production < −50).^2^ Twelve controls were collected separately but their language lateralization was assessed using the same fMRI paradigm. The 12 controls each had a strongly leftward HFLI score (>50). HFLI distributions for RHLD and controls subjects are shown in Figure 2, and information on sex is given in Table 1. The median age of RHLD subjects was 24.5 years, range 20 to 29 years, and for controls the median age was 19 years, range 18 to 24 years. All control subjects were right‐handed in the GOAL data set as assessed by the Edinburgh inventory.^36^

Informed consent was obtained from all participants, and ethical approval for the study was obtained from the Ethics Committee of the Ghent University Hospital.

## 4 WHOLE GENOME SEQUENCING, PRE‐PROCESSING AND VARIANT CALLING

### 4.1 BIL&GIN

Whole genome sequencing (WGS) of the 39 BIL&GIN subjects was performed using Illumina's HiSeq technology by the genomics research organization and service company BGI (Hong Kong/Shenzhen) (https://emea.illumina.com/systems.html). Thirteen additional subjects of European descent, who were not part of the present study, were also sequenced at the same time, and their data processed together with the 39 through preprocessing and variant calling stages (as some of the processing steps below benefit from being run on the greatest sample size available; a minimum of 30 is recommended^38^).

Sequencing was performed at 20 times average coverage depth, with 90 base pair (bp) paired‐end reads for 11 of the RHLD subjects and 14 controls, and 150 bp paired‐end reads for six RHLD subjects and eight controls. Raw reads were cleaned by excluding adapter sequences, reads with low‐quality bases for more than 50% of their lengths, and reads with unknown bases for more than 10% of their lengths. Clean reads were mapped onto the human reference genome (hg19) using the software Burrows‐Wheeler Aligner.^39^ Bam files were sorted using SAMtools v1.2 40 and polymerase chain reaction (PCR) duplicate reads were marked using Picard v1.134. Re‐alignment around indels (insertion/deletions), and base quality control recalibration was performed using the Genome analysis toolkit software (GATK v3.5).^41^, ^42^ Genetic variants were called using the HaplotypeCaller (HC) tool of GATK (v3.5). HC was run separately per sample using the “‐ERC GVCF” mode, and then merged together using the GenotypeGVCFs tool, as recommended in the GATK best practices. We performed Variant Quality Score Recalibration (VQSR) to exclude low quality variants (phred‐scaled Qscore <30) and to flag the rest into the sensitivity tier they fell into (90, 99, 99.9 and 100).

These variants were then normalized, and variants belonging to any VQSR sensitivity tier over 99% were excluded. For the 39 BIL&GIN subjects of this study, the variant calling of SNPs and indels identified on average 4 165 806 variants per subject for the 90 bp protocol (range: 4 079 049‐4 330 101), and 4 484 638 per subject for the 150 bp protocol (range: 4 354 345‐4 657 333).

## 5 GOAL

The genomics company Novogene (Hong Kong/Shenzhen) performed WGS on the 28 samples of the GOAL data set using Illumina's HiSeq Xten technology, and paired‐end sequencing with reads of 150 base pairs and 30x sequence depth. The same pipeline as that applied to the BIL&GIN data was used for alignment (build 37), variant calling, annotation and filtering (but updated to SAMtoolsv1.3.1, Picard v2.0.1, GATK v4.0.1.1 and Gemini v20.0.1, as sequencing of the GOAL subjects was performed later). The variant calling and VQSR steps were carried out together with data from 34 European‐descent subjects who were not part of the present study, again because these steps benefit from a larger number of subjects. These variants were then normalized using the software tool vt normalize (v0.5772‐60f436c3)^43^ and variants belonging to any VQSR sensitivity tier over 99% were excluded. This process resulted in an average of 4 518 323 SNPs and indels per subject (range: 4 318 448‐4 701 297).

### 5.1 Stratification and inbreeding

Within the BIL&GIN and GOAL data sets separately, population structure was assessed by calling genotypes from the sequence data for selected sets of common variants (BIL&GIN: 77 553 variants, GOAL: 41 273 variants) spanning the autosomes. These were high‐confidence single‐nucleotide polymorphism (SNP) sites identified by the 1000 Genomes Project, 1000G_phase1.snps.high_confidence.hg19.vcf.gz with minor allele frequencies (MAFs) > 10% in each data set,^38^ and had been pruned to be in low linkage disequilibrium (LD) with one another using the program PLINK (v1.9) (maximum LD r‐square 0.2).^44^, ^45^ Multidimensional scaling was used to visualize the major dimensions of genome‐wide variability (Figure S1). None of the first five dimensions was associated with the RHLD vs control distinction in either of the data sets (all |T| < 1, *P* > .33). Inbreeding was assessed with the F coefficient estimate within each data set using PLINK (v1.9).^45^ The measure was not associated with the RHLD vs control distinction in either data set (both |T| < 1, *P* > .39).

Note that common genetic variants were only used for the purposes of assessing population stratification and inbreeding within the data sets, whereas the rest of the study was focused on rare genetic variation, which has the potential to involve highly penetrant effects.

### 5.2 Annotation of SNPs and indels

SNPs and indels were annotated using Annovar^46^ and Variant Effect Predictor (v88).^47^ In the genome, nonsynonymous protein‐coding variants, and variants which affect splice donor and acceptor sites, are a priori the most likely to grossly alter gene function. Accordingly, Gemini (v.20.0)^48^ was used to select protein coding variants with “MEDIUM” or “HIGH” impact severity annotations, as well as noncoding variants with “HIGH” impact severity annotations (in practice those altering splice donor or acceptor sites). Additional filtering was performed in R and comprised the removal of “MEDIUM” variants with a PolyPhen^49^ prediction score of “benign”. MAF information was assigned as the maximum MAF across the GNOMAD (v1), ExAC (v3), 1KG, and ESP data sets (ie, “max_aaf_all” in Gemini), which together comprise whole exome or whole genome data from more than 120 000 people from various population data sets^50^ (http://evs.gs.washington.edu/EVS/, http://www.internationalgenome.org/home). Within the BIL&GIN and GOAL data sets separately, any variants present in at least 19 participants (case or control) were excluded as they are likely to be platform‐specific errors or else common variants not previously detected by other sequencing platforms or protocols, and would necessarily be present in at least two control subjects in BIL&GIN or three controls in GOAL (hence unlikely to be high‐penetrance mutations for RHLD).

### 5.3 Monogenic mutation models

*Recessive*: Here, we considered only homozygous or compound heterozygous mutations as potentially trait‐causal. For screening purposes, compound heterozygosity was assigned when a given gene had at least two different mutations, although allelic phase information was not usually available due to the limited sequence read lengths. Variants were excluded when they had MAF ≥ 10% on the basis of on‐line population databases (see above). At 10% MAF, assuming Hardy‐Weinberg equilibrium, the variant would be present in homozygous form at 1% in the population, that is, roughly equal to the RHLD frequency in the population. In the case that 50% penetrance might arise from L‐R randomization, as has been observed for mutations which cause *situs inversus* with PCD,^51^ it is theoretically possible that a single causal variant in a gene could have up to 14% population frequency under a recessive model and Hardy‐Weinberg equilibrium, and still be consistent with a trait frequency of 1%, if it was the only variant involved and caused all cases of the trait. However, allelic and genetic heterogeneity are typical for monogenic traits. Therefore a MAF threshold of 10% under a recessive model is an inclusive rather than strict filter. Variants not present or with no MAF information in the population databases were retained. There were on average 43 recessively mutated genes per subject for the BIL&GIN‐90 bp protocol (range: 31‐61), 64 per subject for the BIL&GIN‐150 bp protocol (range: 55‐77), and 45 per subject for the GOAL data set (range: 33‐64). Integrative Genome Viewer (IGV v2.3.55) was used to visualize the possible compound heterozygous mutations, and genes carrying these were discarded when both mutations were definitely present on the same allele (ie, “in phase”) on a given sequence read.

*Dominant*: Here, we considered heterozygous or homozygous mutations as potentially trait‐causative. Variants were excluded as potentially causative when they had population MAF ≥ 1% in the population databases, on a similar logic as for the recessive model above, but appropriate for allelic dominance and the frequency of RHLD in the population (roughly 1%). Variants not present or with no MAF information in the population databases were retained. There were on average 196 genes per subject for the BIL&GIN‐90 bp protocol (range: 154‐215), 240 per subject for the BIL&GIN‐150 bp protocol (range: 208‐268), and 262 per subject for the GOAL data set (range: 229‐300).

### 5.4 Gene‐level testing

The BIL&GIN and GOAL data sets were combined for subsequent analysis.

We first verified that the total number of mutated genes per subject did not differ significantly between RHLD and control subjects, under either the dominant or recessive model (*t* tests, all *P* > .10). Significance for single‐gene analysis was then assessed separately for individual genes and models (recessive or dominant), using the one‐tailed Fisher's exact test for a 2 × 2 contingency table, for the categories “mutated” and “not mutated” in 33 RHLD subjects and 34 controls. The minimum number of mutated RHLD subjects to achieve a nominally significant *P* value (ie, less than .05) was 5, that is, if a gene would be mutated in five out of the 33 RHLD subjects and none of the 34 controls, that gene would show a nominally significant *P* value of association with RHLD, as a putative major‐genetic effect (*P* value = .0267). This approach allows for allelic heterogeneity, that is, the unit of testing is the gene, within which a variety of different mutations can be present. Note that the power and sample size considerations when modeling highly penetrant effects are different to typical genome‐wide association studies of common traits, in which large samples are screened for common variants of small effect. Here, we focus only on rare variants and interrogate the data with respect to the possibility of high penetrance. Note also that the Fisher's exact test is robust for the sample size, because the significance is assessed with respect to all of the actual possibilities that might have arisen in the contingency table in this set of subjects.

We calculated that for an individual gene to be significant at *P* < .05 after Bonferroni multiple testing correction, it would have to be mutated in at least 11 (dominant) or 10 (recessive) of the 33 RHLD subjects, and no controls, leading to nominal *P* = .000186 (dominant) or *P* = .000373 (recessive) in the Fisher's exact test, that is, the gene would need to be a monogenic cause for roughly one third of the instances of RHLD. For these calculations, we counted how many individual genes, y, have mutations in at least x subjects, for every value of x from 1 to 67 subjects. For each value of x, we then calculated the minimum number of RHLD subjects with mutations in a given gene that would be required to produce a *P* value less than .05/y in the Fisher's exact test.

We performed a post hoc filtering step in which we further excluded from consideration, as potentially monogenic effects, all genes which were mutated in at least one control subject, as these genes were unlikely to be causal monogenically for RHLD. Note that this filter was only applied after the statistical analysis, in order not to bias the multiple testing correction.

### 5.5 Mutational load in gene sets

We tested whether the RHLD cases had an increased mutational load in specific candidate gene‐sets (see the Introduction for the rationale). These candidate sets, based on the gene ontology (GO) as defined within AmiGO's direct annotation^52^, ^53^ (http://geneontology.org/gene-associations/goa_human.gaf.gz downloaded 16‐Nov‐2017), were “cilium” (GO:0005929), “left‐right axis specification” (GO:0070986), “actin cytoskeleton” (GO:0015629), plus two sets defined on the basis of visceral laterality phenotypes or disorders: 58 genes related to PCD and asymmetry disorders^18^; 62 genes either implicated in visceral asymmetry disorders or known to be involved in the visceral left‐right developmental pathway,^20^ as well as a final set of 18 candidate genes which have been tentatively associated with human brain laterality in previous studies.^14^, ^30^, ^32^

The GO terms were defined within AmiGO's^52^, ^53^ direct annotation (http://geneontology.org/gene-associations/goa_human.gaf.gz, downloaded 16‐Nov‐2017). Additional sets were investigated post hoc as child sets of the actin cytoskeleton set (Table S2). Only gene sets comprising at least 10 genes were considered.

To test for an increased mutational load within a given gene‐set in RHLD, the sum of the number of mutated genes (as defined above) per subject within the set was compared between RHLD subjects and controls by means of the one‐tailed exact binomial test, that is, considering the sum of mutated genes per subject in RHLD subjects only, the total sum across RHLD and controls combined, and the proportion of all subjects who were RHLD (33/67). Again, as an exact test, the binomial is robust for the subject sample size, and does not require assumptions on the number of mutations per individual.

### 5.6 Association with handedness within the UK Biobank

Because the large majority of people with RHLD are left‐handed, any monogenic contributions to RHLD would likely also be strongly penetrant for left‐handedness. We checked whether a specific mutation of interest in the gene *TCTN1,* rs188817098, which we initially considered a potential candidate for causing RHLD in some subjects (see Results), is also associated with handedness the UK Biobank cohort data. There were 330 474 subjects (32 367 left‐handed) available for this analysis. In this data set, rs188817098 had been directly genotyped and was in Hardy Weinberg equilibrium (*P* = 1), and the minor allele C had a frequency of 0.001305. Handedness (UK biobank field ID: 1707.0.0) was self‐reported and coded for the present purposes as “left‐handed” or “right‐handed”, as described elsewhere.^54^ We performed association analysis of rs188817098 with handedness using the program BOLT‐LMM (v2.3) which uses linear mixed effects regression under an additive genetic model.^55^ The top 40 principal components capturing genetic diversity in the genome‐wide genotype data, calculated using fastPCA^56^ and provided by the UK biobank,^57^ were included as covariates to control for population structure, as well as sex, age, genotyping array, and assessment center. The UK Biobank data were obtained as part of research application 16 066, with Clyde Francks as the principal applicant. The data collection for the UK Biobank has been described elsewhere.^58^ Informed consent was obtained by the UK Biobank for all participants.

## 6 RESULTS

### 6.1 Monogenic mutational models

We focused on mutations in the 33 RHLD cases which are known to be relatively rare in the general population on the basis of large‐scale genetic databases and predicted to disruptively affect protein sequence, while not being mutated in a set of 34 control subjects (see Methods). As noted above, a given gene would need to be a monogenic cause for at least 10 or 11 of the 33 RHLD cases in this study, and not mutated in controls, to be detected at a significant level after multiple testing correction. There were no genes which met this threshold, under either the dominant or recessive models.

Under a recessive model, no gene was even nominally significant (ie, showed unadjusted *P* < .05), which could have arisen from being mutated in as few as five RHLD cases and no controls.

In the dominant model, *TCTN1* was the only nominally significant gene (*P* = .0267 before multiple testing correction), with five RHLD cases and no controls having heterozygous mutations (Table 2). *TCTN1* encodes a member of a family of secreted and transmembrane proteins and is a component of the tectonic‐like complex, which forms a barrier between the ciliary axoneme and the basal body.^59^ This gene tolerates missense and loss of function variation well (as reflected by the ExAC missense Z‐score^50^: *z* = 0.20). Recessive mutations in *TCTN1* cause Joubert syndrome (JBTS, MIM #614173), a ciliopathy characterized by cerebellar and brainstem malformations.^59^, ^60^

**Table 2:** All putative mutations within *TCTN1*

Chr	Position	Ref	Alt	MAF	RS ID	Impact	AA change	Gemini severity	Poly Phen	Sift	PFAM	CADD	RHLD	Ctrl
12	111 070 349	GA TA	G	3.3E‐3	rs529269328	inframe del	p.N235del	MED	‐	‐	DUF1619	‐	1	0
12	111 078 865	G	C	8.0E‐4	rs201990420	missense	p.V339 L	MED	PosD	D	DUF1619	16.3	1	0
12	111 080 154	G	C	0.0014	rs188817098	missense	p.V431 L	MED	PosD	D	‐	26.1	3	0

*Note*: RS ID refers the variant identity in dbSNP. PolyPhen prediction: PosD: possibly damaging. Sift prediction D: deleterious. PFAM: protein domain. CADD: CADD score v1. The RHLD and Ctrl columns show the numbers of these mutations in cases and controls (all were heterozygous).

Abbreviations: Chr, chromosome; Ref, reference allele; Alt, alternative allele; MAF, maximum minor allele frequency across 1KG, ExAC, gnomAD populations; AA, amino acid.

Three of the five RHLD cases shared the same *TCTN1* missense variant (chr12:111080154 G/C, rs188817098), which has a maximum population frequency of 0.001199 (in ExAC non‐Finnish Europeans). This variant is present in ClinVar (https://www.ncbi.nlm.nih.gov/clinvar/) as a variant of uncertain significance with potential relevance to Joubert syndrome/Meckel‐Gruber syndrome patients (SCV000634600.1). The other two *TCTN1* mutations were a missense variant (chr12:111078865 G/C, rs201990420) and an in‐frame deletion (chr12:111070349 GATA/G), each present in one RHLD case only, and with maximum population frequencies of 0.0008 and 0.0033, respectively.

Rs188817098 was also associated with handedness in the UK biobank data set (*P* = .034), with the minor allele C (frequency 0.001305) associated with left‐handedness (odd ratio = 1.24). However, this modest effect does not seem compatible with a role of this variant as a highly penetrant cause of RHLD and left‐handedness.

### 6.2 Gene‐set analysis

We analyzed a small number of candidate gene sets involved either in visceral laterality or else the actin cytoskeleton (see Introduction for the rationale). We observed an enrichment of mutations within the ‘actin cytoskeleton’ (GO:0015629) gene‐set (Table 3). This gene set comprises 205 human genes (Table S1) which contribute to the actin cytoskeleton, that is, the internal framework of the cell, composed of actin and associated proteins. Within the genomes of the 67 participants of this study, there were 171 different mutations present in 92 genes belonging to this set. About 59.6% of the instances of mutated genes (102 out of 171) were in the subjects with RHLD, whereas the null probability of a mutated gene falling in a subject with RHLD was 49.25% (ie, 33/67), exact binomial test *P* = .0040 (Table 3 and Figure S3). This suggests that individuals with RHLD have a significant enrichment of rare, disruptive mutations in genes involved in actin cytoskeleton structure and function.

**Table 3:** Mutation load analysis of candidate gene sets

Gene set	Set size	GO ID	RHLD	Total	*P*
Actin cytoskeleton	205	GO:0015629	102	171	.004048
Cilium	173	GO:0005929	86	177	.60
Left/right axis specification	13	GO:0070986	6	13	.69
Reiter & Leroux^18^	58	‐	25	49	.46
Deng et al^20^	63	‐	29	60	.61
Francks^14^Gunturkun & Ocklenburg^32^de Kovel & Francks^30^	18	‐	21	41	.46

*Note*: Set size: number of genes within set. RHLD: instances of genes carrying mutations within RHLD cases; Total: instances of genes carrying mutations in RHLD cases and controls combined. The *P*‐value is shown from the exact binomial test, where the null probability was .493 (33/67 participants being RHLD) and alternative hypothesis = “greater”. Reiter & Leroux (2017): 58 genes related to primary ciliary dyskinesia and asymmetry disorders. Deng et al (2015): 62 genes either implicated in visceral asymmetry disorders or known to be involved in the visceral left‐right developmental pathway. Francks (2015), Gunturkun & Okclenburg (2017), de Kovel & Francks (2018): 18 genes previously associated with brain/behavioral laterality phenotypes in humans.

In contrast, no differences were found between participants with RHLD and controls for the GO sets “cilium” (GO:0005929), “left‐right axis specification” (GO:0070986), or sets defined on the basis of visceral laterality phenotypes or disorders,^18^, ^20^ as well as the set of 18 candidate genes which have been tentatively associated with human brain laterality in previous studies (Table 3), consistent with language dominance being largely or wholly independent of these pathways/sets.

We investigated subsets of genes defined as belonging to specific components of the actin cytoskeleton, which included “actin filament” (GO:0005884), “myosin complex” (GO:0016459), and “cortical actin cytoskeleton” (GO:0030864), but saw no significant increase in mutation rates in RHLD in these sets (Table S2). This may indicate that subsets of actin cytoskeleton genes that are more specifically relevant to lateralized brain development have not been defined within the GO.

Post hoc analysis of mutational load within the actin cytoskeleton gene set was further performed in different subsets of subjects according to handedness: RHLD vs right‐handed controls only (*P* = .04), RHLD vs left‐handed controls only (*P* = .004), right‐handed controls vs left‐handed controls (*P* = .88) (Table S3). This pattern indicates that left‐handedness without RHLD is not linked to an increased rate of mutations in actin cytoskeleton genes, and that the tentative increase was a specific property of the RHLD subjects.

Per data set analysis showed that the increased mutational load in the actin cytoskeleton gene set was mostly driven by the BIL&GIN data set (*P* = .0006), while the effect was not significant in the GOAL data set (*P* = .4) despite having a similar trend of increased mutational load in RHLD cases (Table S4, Figure S3).

## 7 DISCUSSION

Laterality is an important feature of the human brain's structural and functional organization.^14^, ^61^, ^62^ Despite this, very little is known of the genetic contributions to typical brain laterality and its variation. In the present study, we performed the first molecular genetic investigation of RHLD, a trait which is present in only roughly 1% of the population. We focused on relatively rare coding variants that are predicted to disrupt protein functions. A highly penetrant mutated gene in roughly one‐third of the RHLD cases, and no controls, could have been detected at a significant level after adjusting for multiple testing in this study. This is a similar level of genetic heterogeneity as found in situs inversus of the visceral organs when it occurs together with PCD, for which up to roughly one quarter of cases are due to mutations in a single gene, *DNAH5* 19.

However, we found no individual genes mutated in RHLD at this level, in the present study. It remains possible that some monogenic causes of RHLD were present in our data set, but we could not distinguish them with the present sample size. Note that the sample size precluded an investigation of common genetic effects with low penetrance, that is, the kinds of effects that are tested in typical genome‐wide association studies of common traits. The approach here was necessarily focused only on rare variants, which might have sometimes acted as highly penetrant mutations. Nonetheless, it appears on the basis of our data that substantial genetic heterogeneity is likely to be involved in any heritable contribution to RHLD, even if some individual effects might be strongly penetrant. As noted in the introduction, non‐leftward language dominance has previously been shown to have a heritability of roughly 30%, although the trait definition in that study included ambilateral individuals in addition to RHLD.^10^

As RHLD is mostly found in left‐handed people,^3^ and comprises roughly 10% of the left‐handed population, then any highly penetrant genetic effects on RHLD would presumably also be strongly associated with left‐handedness. One individual gene, *TCTN1*, carried rare, protein‐altering mutations in five RHLD cases and no controls. Three of these cases carried the same rare variant, and the very large UK Biobank data set, comprising hundreds of thousands of participants, allowed us to test this rare variant for association with left‐handedness. (No functional imaging measures of language laterality were available in the UK Biobank to study RHLD in that data set.) Although the *TCTN1* variant showed a significant association with left‐handedness, in the expected direction (ie, the minor allele associated with left‐handedness), the effect size was not compatible with a highly penetrant effect. Therefore, this finding remains ambiguous.

In the present study, candidate genes that have been tentatively associated with human brain laterality in previous studies showed no evidence for an increase in mutation load in RHLD. The only gene among these that had more mutations in RHLD cases than those in controls was *AR* (eight in RHLD cases, six in controls). For most of these genes, there is no clear mechanism that might link them to left‐right axis determination through chiral properties.

We also found no evidence that candidate gene sets involved in visceral laterality or PCD have an enrichment of rare, protein‐altering mutations in RHLD. This finding is consistent with the fact that people with *situs* inversus of the viscera, when it occurs together with PCD, have shown normal population rates of left‐handedness and left hemisphere language dominance.^21^, ^22^, ^23^ Therefore, there appears to be a developmental disconnect between nodal‐ciliary‐induced visceral laterality and the functional brain lateralities for hand dominance and language. This suggests that at least some aspects of human functional brain laterality arise from an independent and unknown mechanism, which may be brain‐intrinsic. A molecular‐developmental pathway for laterality in the zebrafish brain has been relatively well described, but this appears to take its original cues from the nodal‐visceral pathway, and thus the relevance for human functional brain laterality is not clear.^63^, ^64^ A relatively small‐scale genome‐wide association study in humans reported that genes involved in visceral laterality showed an enrichment of association signals with left‐vs‐right hand motor skill,^65^ but a much larger study of binary‐trait handedness in the UK Biobank data set, based on roughly 350 000 subjects, found no genetic link of handedness to visceral asymmetry genes.^30^ Early life factors can also influence handedness, including birth weight, twinning and breastfeeding, but to an extent which is not remotely predictive at the individual level.^54^

Intriguingly, it may be that situs inversus of the visceral organs does associate with left‐handedness when not due to mutations affecting the nodal ciliary pathway,^23^ although no causal genes were identified in a recent study which investigated the trait combination of situs inversus and left‐handedness without PCD.^66^ Here, we found initial evidence that people with RHLD have an elevated rate of rare, protein‐altering mutations in genes involved in the structure and function of the actin cytoskeleton. This effect was robust to the use of either left or right‐handed control groups, and thus was a specific property of RHLD subjects in this data set, rather than left‐handedness in general. We speculate that functional language laterality may be grounded in an evolutionarily ancient mechanism of inducing organ‐intrinsic left‐right morphogenesis, which can be traced back to the ancestral bilateria, and which arises from fundamental aspects of cellular biology and mechanics.^24^, ^25^, ^27^ Developmental studies will be needed to assess whether cellular chirality is transiently present before asymmetric embryonic development of the mammalian brain. An understanding of how mutations of actin cytoskeleton genes might affect such a process will depend on detailed analysis of cellular models. An increased load of heterozygous mutations in genes affecting the actin cytoskeleton might affect brain laterality, while being otherwise well tolerated during development, due to compensation by non‐mutated alleles at most of the genes involved. Given that common variants of the microtubule‐associated gene *MAP2* have recently been associated with left‐handedness by large‐scale GWAS,^30^, ^31^ our findings here in relation to RHLD may be broadly concordant, insofar as they also implicate the cytoskeleton in the developmental origins of human brain laterality.

The possible link of RHLD to actin cytoskeleton genes will need to be replicated in larger independent data sets. Within the present study, we combined the BIL&GIN and GOAL data sets to maximize the power to detect genetic effects on RHLD, although the functional tasks used to define RHLD differed between these two data sets: hemispheric dominance was defined using a contrast at the sentence level in BIL&GIN, and a word‐level contrast in GOAL (see Methods). However, we are not aware of a large‐scale data collection in existence, or currently underway, in which a harmonized phenotypic measure of RHLD will become available and which would be well‐powered for GWAS.

Given the sample size for the present study, we focused on rare, protein‐altering mutations which had the potential to be highly penetrant effects. Whole genome sequence data, of the type produced in the present study, also contain information on noncoding variation. Rare noncoding variation has recently been implicated in neurodevelopmental disorders such as autism,^67^, ^68^ and a significant fraction of this variation is potentially important for gene function and regulation.^69^ The noncoding genome comprises 98% of the genome, and interpreting the variation within these regions is challenging. Several attempts have been made to rank potentially causative variants across the genome based on scores that integrate different types of information, including conservation of DNA sequence, regulatory information,^70^ and population genomic data. These ranking approaches include CADD,^71^ DANN,^72^ GWAVA,^73^ M‐CAP,^74^ MetaSVM^75^ or REVEL.^76^ However, these ranking approaches are not very concordant with each other.^69^ Moreover, the methods rely on assumptions about the deleteriousness/pathogenicity of variants, so that the overall approach is not an obvious fit for a non‐pathogenic trait such as RHLD. Thus we did not pursue investigation of non‐coding variation, which must await larger sample sizes and an improved understanding of the role of rare, non‐coding variation in non‐disease phenotypic variation.

Data sets based on hundreds of thousands of participants, such as the UK biobank,^77^ permit the estimation of how much of the variance in brain traits can be explained by common genetic variants, and the detection of genetic loci with very small effect sizes. However, the use of such large data sets is usually at the expense of detailed and accurate phenotypic characterization. Correlated structural^78^ or resting‐state derived indices^79^ may offer alternative ways to study RHLD in large data sets, but these approaches will always be indirect. Hence, the approach taken in the present study is complementary to large‐scale studies. We expect that convergent evidence arising from different strategies will help us better understand the biological underpinnings of language lateralization.

## CONFLICT OF INTEREST

The authors declare no potential conflict of interest.

## Supporting information


**Table S1.** All putative mutations in actin cytoskeleton genes. Chr, chromosome; Ref, reference allele; Alt, alternative allele; MAF, maximum minor allele frequency across 1KG; ExAC, gnomAD populations; RS ID, refers the variant identity in dbSNP; AA, amino acid; PrD, probably damaging; PosD, possibly damaging. The RHLD and Ctrl columns show the numbers of these mutations in cases and controls (all were heterozygous). The 113 remaining genes in the actin cytoskeleton gene set, which had no mutations in this data set, were as follows: *ABCB4, ABL2, ACACA, ACTA2, ACTB, ACTR2, ACTR3, ADAM17, AIF1L, ALDOA, AMPH, ANLN, ARC, ARHGAP21, ARHGAP35, ARHGAP6, ARPC1A, ARPC2, ARPC3, AUTS2, BAIAP2, BAIAP2L1, BAIAP2L2, BIN1, C10orf90, CALD1, CAPZA1, CAPZA2, CAPZB, CARMIL2, CASK, CATIP, CD2AP, CDC42EP3, CDH1, CENPQ, CFL2, CLIC4, CNN3, CNR1, CORO1C, CORO2A, CORO2B, CORO6, CRK, CTDP1, CTNNA2, DAPK1, DDX58, DHX9, DMTN, DSTN, FGR, FOXA3, FSCN1, FYB1, GABARAP, GAS2L3, H1F0, HAP1, HAX1, HNRNPC, HSPB7, IFIT5, INTS6, IPP, IQGAP2, IVNS1ABP, KANSL2, KLHL14, KLHL2, KLHL20, MARCKS, MPRIP, MSRB1, MYL2, MYLK3, MYOT, MYOZ1, MYOZ2, MYOZ3, NCOA5, ONECUT2, OPHN1, PGM1, PKNOX2, POU6F1, PPP1R12A, RAB22A, RAB5A, RARA, RND1, SEPT2, SEPT9, SORBS2, STK17B, STK38L, STOML2, TAF5, TARS, TAX1BP3, TLN2, TMEM63B, TOPBP1, TPM1, TPM2, TPM3, TRMT10A, TTC17, USH1G, WAS, WIPF1, ZNF174*.
**Table S2.** Mutation analysis of GO:0015629 “actin cytoskeleton” in relation to its child sets, and negative control gene sets of similar sizes to GO:0015629. Set size: number of genes within set. RHLD: instances of genes carrying mutations within RHLD cases; Total: instances of genes carrying mutations in RHLD cases and controls combined. The *P*‐value is shown from the exact binomial test, where the null probability was .4925 (33/67) participants being RHLD) and alternative hypothesis = “greater”. Negative control sets were identified which had set sizes similar to the actin cytoskeleton set, and no known links to the biology of left‐right asymmetry.
**Table S3.** Mutation analysis of GO:0015629 “actin cytoskeleton” by different handedness groups. Test: instances of genes carrying mutations within the test group; Total: instances of genes carrying mutations in the test and contrast groups. Proportion = Test/Total. Null: null probability, given the proportion of test subjects for the contrast being tested. The *P*‐value is shown from the exact binomial test, with the alternative hypothesis = “greater”.
**Table S4.** Mutation analysis of GO:0015629 “actin cytoskeleton” within each data set. Test: instances of genes carrying mutations within the test group; Total: instances of genes carrying mutations in the test and contrast groups. Proportion = Test/Total. Null: null probability, given the proportion of test subjects for the contrast being tested. The *P*‐value is shown from the exact binomial test, with the alternative hypothesis = “greater”.
**Figure S1.** Multidimensional scaling (MDS) to capture overall genomic diversity among the study samples (black squares = RHLD cases, black triangles = controls), in relation to the 1000 Genomes populations of known geographic ancestries (dots). 1KG super population codes: AFR, African; EAS, east asian; AMR, Ad Mixed American; EUR, European.
**Figure S2.** Histogram distribution of the manual preference strength variable assessed by the Edinburgh Handedness Inventory (EHI) score across RHLD cases and controls, per data set.
**Figure S3.** Distribution of mutations within the “actin cytoskeleton” gene set.
