# Supplementary material for: Genome sequencing for rightward hemispheric language dominance
Source: Genes Brain Behav. 2019 Apr 23;18(5):e12572. doi: 10.1111/gbb.12572 (PMC6850193; doi:10.1111/gbb.12572)
Supplement: Supplementary file 1 — Table S1. All putative mutations in actin cytoskeleton genes. Chr, chromosome; Ref, reference allele; Alt, alternative allele; MAF, maximum minor allele frequency across 1KG; ExAC, gnomAD populations; RS ID, refers the variant identity in dbSNP; AA, amino acid; PrD, probably damaging; PosD, possibly damaging. The RHLD and Ctrl columns show the numbers of these mutations in cases and controls (all were heterozygous). The 113 remaining genes in the actin cytoskeleton gene set, which had no mutations in this data set, were as follows: ABCB4, ABL2, ACACA, ACTA2, ACTB, ACTR2, ACTR3, ADAM17, AIF1L, ALDOA, AMPH, ANLN, ARC, ARHGAP21, ARHGAP35, ARHGAP6, ARPC1A, ARPC2, ARPC3, AUTS2, BAIAP2, BAIAP2L1, BAIAP2L2, BIN1, C10orf90, CALD1, CAPZA1, CAPZA2, CAPZB, CARMIL2, CASK, CATIP, CD2AP, CDC42EP3, CDH1, CENPQ, CFL2, CLIC4, CNN3, CNR1, CORO1C, CORO2A, CORO2B, CORO6, CRK, CTDP1, CTNNA2, DAPK1, DDX58, DHX9, DMTN, DSTN, FGR, FOXA3, FSCN1, FYB1, GABARAP, GAS2L3, H1F0, HAP1, HAX1, HNRNPC, HSPB7, IFIT5, INTS6, IPP, IQGAP2, IVNS1ABP, KANSL2, KLHL14, KLHL2, KLHL20, MARCKS, MPRIP, MSRB1, MYL2, MYLK3, MYOT, MYOZ1, MYOZ2, MYOZ3, NCOA5, ONECUT2, OPHN1, PGM1, PKNOX2, POU6F1, PPP1R12A, RAB22A, RAB5A, RARA, RND1, SEPT2, SEPT9, SORBS2, STK17B, STK38L, STOML2, TAF5, TARS, TAX1BP3, TLN2, TMEM63B, TOPBP1, TPM1, TPM2, TPM3, TRMT10A, TTC17, USH1G, WAS, WIPF1, ZNF174. Table S2. Mutation analysis of GO:0015629 “actin cytoskeleton” in relation to its child sets, and negative control gene sets of similar sizes to GO:0015629. Set size: number of genes within set. RHLD: instances of genes carrying mutations within RHLD cases; Total: instances of genes carrying mutations in RHLD cases and controls combined. The P‐value is shown from the exact binomial test, where the null probability was .4925 (33/67) participants being RHLD) and alternative hypothesis = “greater”. Negative control sets were identified which had set sizes similar to the actin cytoskeleton set, and no known links to the biology of left‐right [file GBB-18-na-s001.docx]

**Supplementary information – Carrion-Castillo *et al.*, Genome sequencing for rightward hemispheric language dominance**

# Supplementary Tables

### Table S1: All putative mutations in actin cytoskeleton genes. Chr: chromosome. Ref: Reference allele. Alt: Alternative allele. MAF: maximum minor allele frequency across 1KG, ExAC, gnomAD populations. RS ID refers the variant identity in dbSNP. AA: amino acid. PrD: probably damaging, PosD: possibly damaging. The RHLD and Ctrl columns show the numbers of these mutations in cases and controls (all were heterozygous). The 113 remaining genes in the actin cytoskeleton gene set, which had no mutations in this dataset, were: *ABCB4, ABL2, ACACA, ACTA2, ACTB, ACTR2, ACTR3, ADAM17, AIF1L, ALDOA, AMPH, ANLN, ARC, ARHGAP21, ARHGAP35, ARHGAP6, ARPC1A, ARPC2, ARPC3, AUTS2, BAIAP2, BAIAP2L1, BAIAP2L2, BIN1, C10orf90, CALD1, CAPZA1, CAPZA2, CAPZB, CARMIL2, CASK, CATIP, CD2AP, CDC42EP3, CDH1, CENPQ, CFL2, CLIC4, CNN3, CNR1, CORO1C, CORO2A, CORO2B, CORO6, CRK, CTDP1, CTNNA2, DAPK1, DDX58, DHX9, DMTN, DSTN, FGR, FOXA3, FSCN1, FYB1, GABARAP, GAS2L3, H1F0, HAP1, HAX1, HNRNPC, HSPB7, IFIT5, INTS6, IPP, IQGAP2, IVNS1ABP, KANSL2, KLHL14, KLHL2, KLHL20, MARCKS, MPRIP, MSRB1, MYL2, MYLK3, MYOT, MYOZ1, MYOZ2, MYOZ3, NCOA5, ONECUT2, OPHN1, PGM1, PKNOX2, POU6F1, PPP1R12A, RAB22A, RAB5A, RARA, RND1, SEPT2, SEPT9, SORBS2, STK17B, STK38L, STOML2, TAF5, TARS, TAX1BP3, TLN2, TMEM63B, TOPBP1, TPM1, TPM2, TPM3, TRMT10A, TTC17, USH1G, WAS, WIPF1, ZNF174*.

| Gene | Chr | Position | Ref | Alt | MAF | RS ID | Impact | AA change | Gemini severity | Polyphen | RHLD | Cntrl |
| --- | --- | --- | --- | --- | --- | --- | --- | --- | --- | --- | --- | --- |
| *KLHL17* | 1 | 898617 | C | T | - | - | missense | L/F | MED | PosD | 1 | 0 |
| *KLHL17* | 1 | 898752 | C | T | 5.97E-05 | rs758133453 | missense | S/F | MED | PosD | 0 | 1 |
| *SCNN1D* | 1 | 1222519 | C | T | 0.0038 | rs148588796 | stop gained | R/* | HIGH |  | 1 | 0 |
| *SCNN1D* | 1 | 1223412 | AGGCAGGT  GAGGCTGG  GCTGGCAG  GGGGTGCGGG | A | 0.007463 | rs768185716 | splice donor | | HIGH |  | 1 | 0 |
| *SCNN1D* | 1 | 1226797 | C | G | 0.003388 | rs141251506 | missense | A/G | MED | PosD | 0 | 1 |
| *CROCC* | 1 | 17257820 | G | T | 0.003023 | rs142373017 | missense | R/L | MED | PrD | 1 | 0 |
| *CROCC* | 1 | 17270743 | G | A | 0.000669 | rs6684619 | missense | D/N | MED | PosD | 0 | 1 |
| *CROCC* | 1 | 17282616 | G | A | - | - | missense | E/K | MED | PrD | 1 | 0 |
| *WASF2* | 1 | 27744825 | C | G | 0.005155 | rs150650907 | missense | E/Q | MED | unknown | 0 | 2 |
| *NDC1* | 1 | 54238077 | G | A | 0.003 | rs116087466 | missense | T/I | MED | PrD | 0 | 1 |
| *GBP2* | 1 | 89579827 | C | T | 0.005994 | rs147615005 | missense | V/M | MED | PosD | 0 | 1 |
| *CTTNBP2NL* | 1 | 112998611 | G | A | 0.000117 | rs549525235 | missense | S/N | MED | PosD | 1 | 0 |
| *SPTA1* | 1 | 158582633 | A | G | - | - | missense | Y/H | MED | PrD | 1 | 0 |
| *SPTA1* | 1 | 158614141 | G | A | 0.000511 | rs201399968 | missense | R/C | MED | PrD | 1 | 0 |
| *SPTA1* | 1 | 158639328 | C | T | 0.002049 | rs200829664 | missense | R/H | MED | PrD | 1 | 0 |
| *SPTA1* | 1 | 158645955 | T | C | 0.002438 | rs188840804 | missense | Y/C | MED | PrD | 0 | 1 |
| *DDR2* | 1 | 162740272 | C | T | 0.000161 | rs757051385 | missense | P/S | MED | PosD | 0 | 1 |
| *ARPC5* | 1 | 183599656 | A | T | - | - | stop gained | Y/* | HIGH |  | 1 | 0 |
| *ARPC5* | 1 | 183599660 | A | T | - | - | missense | I/N | MED | PrD | 1 | 0 |
| *ACTA1* | 1 | 229568440 | AGGGT | A | - | - | frameshift | TL/X | HIGH |  | 1 | 0 |
| *ACTA1* | 1 | 229568445 | G | GCACT | - | - | frameshift | P/PVX | HIGH |  | 1 | 0 |
| *FMN2* | 1 | 240256071 | A | C | - | - | missense | Q/P | MED | unknown | 1 | 0 |
| *FMN2* | 1 | 240256334 | G | A | - | - | missense | A/T | MED | unknown | 1 | 0 |
| *FMN2* | 1 | 240256668 | A | C | 0.006395 | rs146681532 | missense | K/T | MED | unknown | 1 | 0 |
| *FMN2* | 1 | 240256781 | G | A | 0.001 | rs142335257 | missense | A/T | MED | unknown | 1 | 0 |
| *PFN4* | 2 | 24345324 | C | T | 0.004186 | rs79726112 | missense | E/K | MED | PosD | 0 | 1 |
| *DCTN1* | 2 | 74605239 | T | C | 0.003 | rs566433112 | missense | K/R | MED | PrD | 1 | 0 |
| *ANKRD23* | 2 | 97505302 | G | T | 0.000139 | rs570323112 | missense | P/H | MED | PosD | 0 | 1 |
| *ANKRD23* | 2 | 97505304 | A | C | 0.000134 | rs775808923 | missense | H/Q | MED | PrD | 0 | 1 |
| *NEB* | 2 | 152369326 | A | G | 0.002584 | rs201767727 | missense | M/T | MED | PrD | 1 | 0 |
| *NEB* | 2 | 152382778 | T | C | - | - | splice acceptor | | HIGH |  | 1 | 0 |
| *NEB* | 2 | 152396904 | G | A | 0.003799 | rs767461697 | missense | T/M | MED | PrD | 1 | 0 |
| *NEB* | 2 | 152420387 | G | A | 0.000541 | rs765644258 | missense | R/C | MED | PosD | 0 | 1 |
| *NEB* | 2 | 152484315 | C | T | 0.004019 | rs75639119 | missense | G/S | MED | PrD | 1 | 0 |
| *NEB* | 2 | 152534230 | A | G | 0.0029 | rs201141958 | missense | I/T | MED | PrD | 1 | 0 |
| *NEB* | 2 | 152553170 | T | C | 0.000206 | rs199710125 | missense | N/S | MED | PrD | 0 | 1 |
| *VILL* | 3 | 38039654 | G | C | 0.001 | rs140337038 | missense | E/Q | MED | PosD | 1 | 0 |
| *FLNB* | 3 | 58116478 | C | G | 0.00114 | rs143831841 | missense | F/L | MED | PrD | 0 | 1 |
| *FLNB* | 3 | 58140550 | G | A | 0.000182 | rs750450803 | missense | A/T | MED | PrD | 0 | 1 |
| *FLNB* | 3 | 58156433 | G | A | 3.58E-05 | - | missense | V/M | MED | PrD | 1 | 0 |
| *KALRN* | 3 | 124281690 | C | T | 0.00634 | rs186739814 | missense | L/F | MED | PrD | 1 | 0 |
| *KALRN* | 3 | 124303696 | C | T | 0.003931 | rs56407180 | stop gained | R/* | HIGH |  | 1 | 0 |
| *AFAP1* | 4 | 7774557 | G | A | 0.001398 | rs151145563 | missense | S/L | MED | PrD | 1 | 0 |
| *AFAP1* | 4 | 7788024 | C | T | 0.000116 | rs138842991 | missense | R/H | MED | PrD | 1 | 0 |
| *AFAP1* | 4 | 7845007 | C | G | - | - | missense | K/N | MED | PosD | 1 | 0 |
| *ABLIM2* | 4 | 8108316 | G | A | 0.00021 | rs752430305 | missense | T/M | MED | PosD | 1 | 0 |
| *CORIN* | 4 | 47680024 | G | T | - | - | missense | P/T | MED | PrD | 1 | 0 |
| *NPFFR2* | 4 | 72994471 | ATCT | A | 0.000348 | rs760474427 | inframe deletion | IF/I | MED |  | 0 | 1 |
| *SNCA* | 4 | 90757941 | C | CT | - | rs3833895,  rs71594901 | splice acceptor | | HIGH |  | 2 | 2 |
| *PDLIM5* | 4 | 95578650 | C | T | 0.005116 | rs148939806 | missense | R/W | MED | PrD | 1 | 0 |
| *SYNPO2* | 4 | 119948129 | C | T | 0.0014 | rs201293157 | missense | S/F | MED | PrD | 0 | 1 |
| *PALLD* | 4 | 169611812 | G | A | 0.004 | rs115372194 | missense | R/H | MED | PosD | 1 | 0 |
| *PALLD* | 4 | 169835099 | C | T | 1.50E-05 | rs758884211 | missense | R/W | MED | PrD | 1 | 0 |
| *PDLIM3* | 4 | 186427772 | C | G | 0.001212 | rs143121072 | missense | V/L | MED | PrD | 1 | 0 |
| *FER* | 5 | 108219171 | T | C | 0.000234 | rs750872853 | missense | L/S | MED | PrD | 0 | 1 |
| *CTNNA1* | 5 | 138145787 | G | A | 8.06E-05 | rs749878552 | missense | R/Q | MED | PrD | 0 | 1 |
| *CTNNA1* | 5 | 138267472 | T | TA | - | - | frameshift | L/LX | HIGH |  | 1 | 0 |
| *APBB3* | 5 | 139940635 | T | C | - | - | missense | Y/C | MED | PrD | 0 | 1 |
| *SYNPO* | 5 | 150029548 | C | T | - | rs571669002 | missense | L/F | MED | PrD | 1 | 0 |
| *SYNPO* | 5 | 150029861 | C | T | 1.80E-05 | - | missense | A/V | MED | PosD | 1 | 0 |
| *SYNPO* | 5 | 150036304 | A | ACCGCCC | - | - | inframe insertion | -/PP | MED |  | 1 | 0 |
| *SYNPO* | 5 | 150036601 | T | TCGGCTCA  AGCGTGGC  AGCCTC | 0.000902 | rs768419688 | inframe insertion | -/RLKRGSL | MED |  | 1 | 0 |
| *DBN1* | 5 | 176887438 | C | G | 0.002865 | rs34301370 | missense | R/P | MED | PrD | 1 | 0 |
| *PDLIM7* | 5 | 176915244 | C | T | 0.002 | rs145726499 | missense | R/H | MED | PrD | 1 | 0 |
| *CLIC5* | 6 | 46047512 | G | T | - | - | stop gained | C/* | HIGH |  | 1 | 0 |
| *FILIP1* | 6 | 76018573 | C | T | 0.000395 | rs147152005 | missense | R/H | MED | PrD | 0 | 1 |
| *WASF1* | 6 | 110434627 | T | C | 0.001047 | rs141069208 | missense | N/S | MED | unknown | 0 | 1 |
| *ULBP1* | 6 | 150290385 | T | C | - | - | missense | W/R | MED | PrD | 1 | 0 |
| *EZR* | 6 | 159188313 | G | A | 0.000136 | rs372964315 | missense | R/C | MED | PrD | 1 | 0 |
| *MAD1L1* | 7 | 2265161 | G | A | 0.005981 | rs121908982 | missense | R/C | MED | PrD | 0 | 1 |
| *MAD1L1* | 7 | 2265186 | C | T | - | - | splice acceptor | | HIGH |  | 0 | 1 |
| *ARPC1B* | 7 | 98991711 | T | G | 0.0029 | rs151225927 | missense | M/R | MED | PosD | 1 | 0 |
| *WASL* | 7 | 123332594 | G | A | 0.000227 | rs371633438 | missense | P/L | MED | unknown | 1 | 0 |
| *FSCN3* | 7 | 127232006 | G | A | 0.0043 | rs138306027 | splice donor | | HIGH |  | 0 | 1 |
| *FSCN3* | 7 | 127240368 | A | G | 0.004583 | rs181240283 | missense | N/S | MED | PosD | 0 | 1 |
| *DENND2A* | 7 | 140267040 | C | T | 0.000515 | rs201765157 | missense | R/Q | MED | PosD | 0 | 1 |
| *MSRA* | 8 | 10159140 | C | T | 6.50E-05 | rs774632520 | missense | P/L | MED | PrD | 0 | 1 |
| *ABRA* | 8 | 107773470 | C | G | 1.50E-05 | rs764154630 | missense | C/S | MED | PrD | 1 | 0 |
| *ABRA* | 8 | 107781766 | C | T | 0.000116 | rs766328261 | missense | R/H | MED | PrD | 1 | 0 |
| *MTSS1* | 8 | 125568190 | G | C | 0.000944 | rs201460545 | missense | P/A | MED |  | 1 | 0 |
| *CCIN* | 9 | 36170317 | G | A | 0.001395 | rs140578907 | missense | R/Q | MED | PrD | 0 | 1 |
| *ACTL7B* | 9 | 111617414 | G | A | 0.002265 | rs115068245 | missense | P/L | MED | PrD | 0 | 1 |
| *GSN* | 9 | 124045671 | G | A | 0.007 | rs192762670 | splice donor | | HIGH |  | 2 | 1 |
| *GSN* | 9 | 124064335 | C | T | 0.000116 | rs149556868 | missense | P/L | MED | PrD | 1 | 0 |
| *ABL1* | 9 | 133760106 | C | T | 0.009304 | rs2229071 | missense | P/L | MED | PrD | 1 | 0 |
| *ABL1* | 9 | 133760600 | C | T | - | - | missense | P/S | MED | PrD | 1 | 0 |
| *SVIL* | 10 | 29779849 | ATCT | A | 6.06E-05 | rs769943300 | inframe deletion | ED/D | MED |  | 0 | 1 |
| *SVIL* | 10 | 29821953 | GCTT | G | 8.96E-06 | - | inframe deletion | EA/A | MED |  | 1 | 0 |
| *SYNPO2L* | 10 | 75407375 | G | A | 8.96E-06 | - | missense | L/F | MED | PrD | 1 | 0 |
| *SYNPO2L* | 10 | 75408353 | C | T | - | - | missense | D/N | MED | PrD | 0 | 1 |
| *NPM3* | 10 | 103543071 | A | G | - | - | missense | L/P | MED | PrD | 0 | 1 |
| *ABLIM1* | 10 | 116222869 | C | A | 0.0014 | rs574961882 | missense | S/I | MED |  | 0 | 1 |
| *ABLIM1* | 10 | 116331054 | A | ACTG | 0.0058 | rs573606552 | splice donor | | HIGH |  | 1 | 0 |
| *LSP1* | 11 | 1887895 | G | A | 0.000404 | rs565801400 | missense | R/Q | MED | unknown | 1 | 0 |
| *LSP1* | 11 | 1888033 | G | A | 3.91E-05 | - | missense | R/Q | MED | unknown | 1 | 0 |
| *AHNAK* | 11 | 62287089 | C | A | 0.000227 | rs199870702 | missense | G/C | MED | PrD | 0 | 1 |
| *AHNAK* | 11 | 62292054 | G | A | 1.80E-05 | - | missense | P/S | MED | PrD | 1 | 0 |
| *AHNAK* | 11 | 62294327 | G | C | 0.0029 | rs143391519 | missense | P/R | MED | PosD | 0 | 1 |
| *AHNAK* | 11 | 62294833 | T | G | 0.001 | rs547487188 | missense | K/N | MED | PrD | 1 | 0 |
| *AHNAK* | 11 | 62297175 | T | C | 0.004115 | rs115693058 | missense | M/V | MED | PrD | 0 | 1 |
| *AHNAK* | 11 | 62297575 | T | G | 0.00093 | rs147401497 | missense | K/N | MED | PrD | 0 | 1 |
| *ARHGAP32* | 11 | 128839094 | A | G | 2.98E-05 | - | missense | L/P | MED | PrD | 1 | 0 |
| *ARHGAP32* | 11 | 128840019 | G | A | 0.0015 | rs142889526 | missense | R/C | MED | PrD | 1 | 0 |
| *TWF1* | 12 | 44200087 | AGCCAGCG  GCCCCG | A | 0.0082 | rs528164053 | frameshift | AGAAG/X | HIGH |  | 0 | 1 |
| *LIMA1* | 12 | 50571079 | C | T | 0.0029 | rs147543382 | missense | G/D | MED | PosD | 1 | 0 |
| *AVIL* | 12 | 58204606 | C | T | 0.008 | rs61938187 | missense | R/H | MED | PrD | 1 | 0 |
| *KNTC1* | 12 | 123032478 | C | T | 0.007562 | rs61751321 | missense | L/F | MED | PrD | 2 | 0 |
| *KNTC1* | 12 | 123068956 | T | C | 0.000602 | rs61751323 | missense | I/T | MED | PrD | 0 | 1 |
| *KNTC1* | 12 | 123089517 | C | A | 0.000133 | rs373134120 | missense | L/M | MED | PrD | 0 | 1 |
| *KNTC1* | 12 | 123107032 | TATC | T | - | - | inframe deletion | I/- | MED |  | 1 | 0 |
| *ZNF268* | 12 | 133779815 | C | T | 0.006073 | rs62644539 | missense | L/F | MED | PrD | 0 | 1 |
| *ZNF268* | 12 | 133780719 | C | G | 0.000127 | rs779235314 | missense | A/G | MED | PosD | 0 | 1 |
| *ZNF268* | 12 | 133780778 | T | C | 4.17E-05 | - | missense | Y/H | MED | PrD | 1 | 0 |
| *FLT1* | 13 | 28896935 | T | G | 0.007 | rs35832528 | missense | E/A | MED | PosD | 0 | 1 |
| *LCP1* | 13 | 46758043 | C | T | - | rs544094121 | splice donor | | HIGH |  | 1 | 0 |
| *SPTB* | 14 | 65253204 | C | T | 0.00093 | rs76283214 | missense | R/H | MED | PrD | 0 | 1 |
| *MYO1E* | 15 | 59430411 | T | C | 0.002144 | rs545492504 | missense | D/G | MED | PosD | 0 | 1 |
| *MYO1E* | 15 | 59487706 | T | G | 0.000605 | rs183502749 | missense | I/L | MED | PosD | 0 | 1 |
| *MYO1E* | 15 | 59497622 | G | C | 0.009847 | rs140447165 | missense | I/M | MED | PrD | 0 | 3 |
| *PEAK1* | 15 | 77406993 | T | C | - | - | missense | I/M | MED | PrD | 0 | 1 |
| *PEAK1* | 15 | 77471361 | G | A | 0.0043 | rs117879553 | missense | R/C | MED | PrD | 1 | 0 |
| *PEAK1* | 15 | 77471383 | C | T | - | - | missense | M/I | MED | PrD | 1 | 0 |
| *IQGAP1* | 15 | 91026731 | C | T | 0.001 | rs140035684 | missense | L/F | MED | PrD | 1 | 0 |
| *BCAR1* | 16 | 75263489 | C | T | 0.000139 | rs745718620 | missense | D/N | MED | PrD | 0 | 1 |
| *BCAR1* | 16 | 75263918 | T | C | 0.001 | rs139024725 | missense | K/E | MED | PrD | 1 | 0 |
| *BCAR1* | 16 | 75269046 | C | T | 0.001632 | rs139270175 | missense | R/Q | MED | PrD | 1 | 0 |
| *SRCIN1* | 17 | 36715877 | G | C | 0.001051 | rs201844245 | missense | L/V | MED | PosD | 1 | 0 |
| *JUP* | 17 | 39932252 | C | T | - | rs544365909 | missense | R/Q | MED |  | 1 | 0 |
| *CDC42EP4* | 17 | 71282144 | G | A | 0.003032 | rs200093546 | missense | R/W | MED | PosD | 0 | 1 |
| *SLC9A3R1* | 17 | 72758167 | G | A | 0.004535 | rs41282065 | missense | R/Q | MED | PrD | 1 | 0 |
| *FSCN2* | 17 | 79496003 | G | A | 0.001775 | rs181961770 | missense | R/Q | MED | PosD | 1 | 0 |
| *FSCN2* | 17 | 79496131 | C | T | 0.000745 | rs377025075 | missense | R/C | MED | PrD | 0 | 1 |
| *C19orf35* | 19 | 2280831 | C | A | 0.000116 | rs371625189 | stop gained | E/* | HIGH |  | 1 | 0 |
| *SPPL2B* | 19 | 2337520 | C | A | 0.000837 | rs201257802 | missense | Q/K | MED |  | 0 | 1 |
| *SPPL2B* | 19 | 2339083 | G | C | - | - | missense | V/L | MED |  | 1 | 0 |
| *SPPL2B* | 19 | 2351565 | C | T | 0.0043 | rs62119750 | missense | A/V | MED |  | 1 | 0 |
| *NOTCH3* | 19 | 15297709 | A | T | 0.001137 | rs148046938 | missense | V/D | MED | PrD | 1 | 0 |
| *NOTCH3* | 19 | 15311618 | TAGC | T | 0.006618 | rs772632118 | inframe deletion | LL/L | MED |  | 1 | 0 |
| *MYO9B* | 19 | 17283662 | T | C | - | - | missense | M/T | MED | PosD | 1 | 0 |
| *MYO9B* | 19 | 17305679 | G | A | 0.005 | rs181046385 | missense | R/H | MED | PrD | 0 | 1 |
| *MYO9B* | 19 | 17320478 | G | A | - | - | missense | S/N | MED | PosD | 1 | 0 |
| *ARHGAP33* | 19 | 36275712 | C | T | 0.006 | rs146584364 | missense | S/F | MED |  | 0 | 1 |
| *ACTN4* | 19 | 39220627 | AGAG | A | 0.000191 | - | splice acceptor | | HIGH |  | 0 | 1 |
| *RINL* | 19 | 39360777 | A | G | - | - | missense | L/P | MED | PrD | 0 | 1 |
| *VASP* | 19 | 46025989 | CAG | C | - | - | frameshift | SG/SX | HIGH |  | 0 | 1 |
| *ZNF74* | 22 | 20761112 | T | A | 0.004678 | rs199810912 | missense | Y/N | MED | PosD | 0 | 1 |
| *SPECC1L* | 22 | 24718408 | G | A | 0.0099 | rs55723436 | missense | R/H | MED | PrD | 2 | 0 |
| *SPECC1L* | 22 | 24720303 | A | G | - | - | missense | D/G | MED | PosD | 1 | 0 |
| *SMTN* | 22 | 31487799 | C | A | 1.19E-05 | - | missense | A/D | MED | PrD | 1 | 0 |
| *SMTN* | 22 | 31500415 | G | A | 0.0014 | rs144949047 | missense | R/Q | MED | PosD | 0 | 2 |
| *MYH9* | 22 | 36685292 | G | A | 0.004651 | rs139134727 | missense | R/W | MED | PrD | 1 | 1 |
| *PDXP* | 22 | 38055250 | TCTC | T | 0.000401 | rs747779022 | inframe deletion | FS/F | MED |  | 1 | 0 |
| *TRIOBP* | 22 | 38121772 | C | T | 3.02E-05 | rs753438438 | missense | A/V | MED | PrD | 1 | 0 |
| *TRIOBP* | 22 | 38129388 | G | A | 0.006815 | rs34066624 | missense | R/Q | MED | PosD | 1 | 0 |
| *TRIOBP* | 22 | 38150924 | G | A | - | - | stop gained | W/* | HIGH |  | 0 | 1 |
| *SHROOM4* | X | 50350726 | T | TC | 0.003202 | rs587780460 | frameshift | E/GX | HIGH |  | 1 | 0 |
| *ZNF185* | X | 152083283 | C | T | 9.96E-05 | rs781893395 | missense | R/C | MED | PrD | 1 | 0 |
| *FLNA* | X | 153581011 | C | A | 0.000464 | rs200524526 | missense | G/C | MED | PrD | 1 | 0 |
| *FLNA* | X | 153587685 | G | A | 0.003556 | rs782426283 | missense | S/L | MED | PosD | 1 | 0 |

### Table S2: Mutation analysis of GO:0015629 'actin cytoskeleton' in relation to its child sets, and negative control gene sets of similar sizes to GO:0015629. Set size: number of genes within set. RHLD: instances of genes carrying mutations within RHLD cases; Total: instances of genes carrying mutations in RHLD cases and controls combined. The P-value is shown from the exact binomial test, where the null probability was 0.4925 (33/67) participants being RHLD) and alternative hypothesis = “greater”. Negative control sets were identified which had set sizes similar to the actin cytoskeleton set, and no known links to the biology of left-right asymmetry.

| Category | Gene set | Set size | GO ID | RHLD | Total | Proportion | Pval |
| --- | --- | --- | --- | --- | --- | --- | --- |
| Hypothesis | actin cytoskeleton | 205 | GO:0015629 | 102 | 171 | 0.597 | **0.004048** |
| child of GO:0015629 | actin filament | 60 | GO:0005884 | 18 | 40 | 0.450 | 0.756484 |
| child of GO:0015629 | Arp2/3 protein complex | 13 | GO:0005885 | 3 | 4 | 0.750 | 0.30139 |
| child of GO:0015629 | myosin complex | 33 | GO:0016459 | 28 | 63 | 0.444 | 0.813034 |
| child of GO:0015629 | cortical actin cytoskeleton | 40 | GO:0030864 | 17 | 39 | 0.436 | 0.807009 |
| child of GO:0015629 | actomyosin | 13 | GO:0042641 | 8 | 15 | 0.533 | 0.476564 |
| negative control | protein polyubiquitination | 221 | GO:0000209 | 57 | 100 | 0.570 | 0.073503 |
| negative control | adaptive immune response | 201 | GO:0002250 | 57 | 113 | 0.504 | 0.436827 |
| negative control | nucleic acid binding | 237 | GO:0003676 | 73 | 133 | 0.549 | 0.112576 |
| negative control | receptor activity | 201 | GO:0004872 | 79 | 159 | 0.497 | 0.488044 |
| negative control | transmembrane signaling receptor activity | 201 | GO:0004888 | 64 | 127 | 0.504 | 0.433079 |
| negative control | DNA repair | 208 | GO:0006281 | 77 | 164 | 0.470 | 0.747769 |
| negative control | rRNA processing | 201 | GO:0006364 | 73 | 138 | 0.529 | 0.220271 |
| negative control | translation | 183 | GO:0006412 | 36 | 67 | 0.537 | 0.270663 |
| negative control | intracellular protein transport | 212 | GO:0006886 | 79 | 147 | 0.537 | 0.157242 |
| negative control | cell surface receptor signaling pathway | 240 | GO:0007166 | 69 | 156 | 0.442 | 0.909195 |
| negative control | keratinization | 182 | GO:0031424 | 96 | 203 | 0.473 | 0.735418 |
| negative control | ion transmembrane transport | 182 | GO:0034220 | 58 | 122 | 0.475 | 0.680229 |
| negative control | Fc-epsilon receptor signaling pathway | 181 | GO:0038095 | 18 | 32 | 0.563 | 0.269502 |
| negative control | leukocyte migration | 206 | GO:0050900 | 59 | 112 | 0.527 | 0.264191 |
| negative control | protein homooligomerization | 202 | GO:0051260 | 63 | 131 | 0.481 | 0.6379 |
| negative control | ubiquitin protein ligase activity | 218 | GO:0061630 | 56 | 105 | 0.533 | 0.230102 |
| negative control | positive regulation of ERK1 and ERK2 cascade | 189 | GO:0070374 | 49 | 92 | 0.533 | 0.253196 |

### Table S3: Mutation analysis of GO:0015629 'actin cytoskeleton' by different handedness groups. Test: instances of genes carrying mutations within the test group; Total: instances of genes carrying mutations in the test and contrast groups. Proportion= Test/Total. Null: null probability, given the proportion of test subjects for the contrast being tested. The P-value is shown from the exact binomial test, with the alternative hypothesis = “greater”.

| Test group | Contrast group | Test | Total | Proportion | Null | Pval |
| --- | --- | --- | --- | --- | --- | --- |
| RHLD | Controls | 102 | 171 | 0.5965 | 0.4925 | 0.004048 |
| RHLD | RH controls | 102 | 147 | 0.6939 | 0.6226 | 0.043420 |
| RHLD | LH controls | 102 | 126 | 0.8095 | 0.7021 | 0.004277 |
| LH controls | RH controls | 24 | 69 | 0.3478 | 0.4118 | 0.885989 |

### Table S4: Mutation analysis of GO:0015629 'actin cytoskeleton' within each dataset. Test: instances of genes carrying mutations within the test group; Total: instances of genes carrying mutations in the test and contrast groups. Proportion= Test/Total. Null: null probability, given the proportion of test subjects for the contrast being tested. The P-value is shown from the exact binomial test, with the alternative hypothesis = “greater”.

| Dataset | Test | Total | Proportion | Null | Pval |
| --- | --- | --- | --- | --- | --- |
| BIL&GIN | 57 | 94 | 0.6064 | 0.4359 | 0.000653 |
| GOAL | 45 | 77 | 0.5844 | 0.5667 | 0.4230 |

# Supplementary Figures


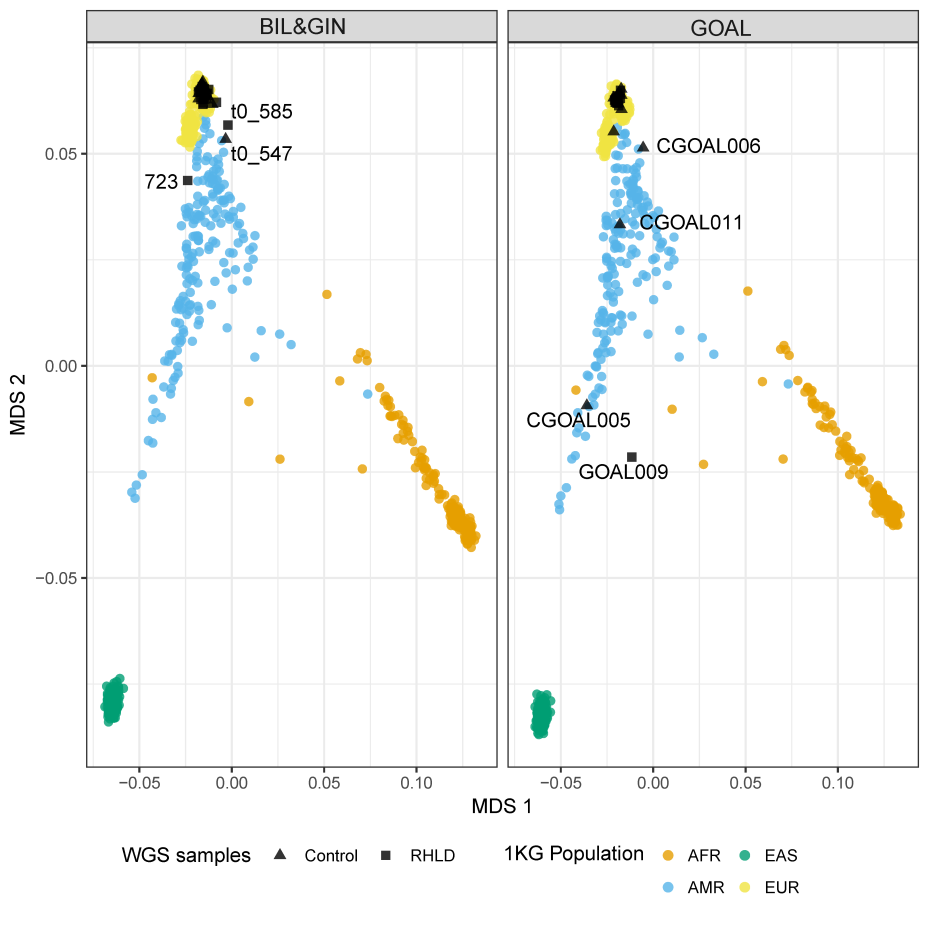


### Figure S1: Multidimensional Scaling (MDS) to capture overall genomic diversity among the study samples (black squares=RHLD cases, black triangles= controls), in relation to the 1000 Genomes populations of known geographic ancestries (dots). 1KG super population codes: AFR: African, EAS: east asian, AMR: Ad Mixed American, EUR: European.


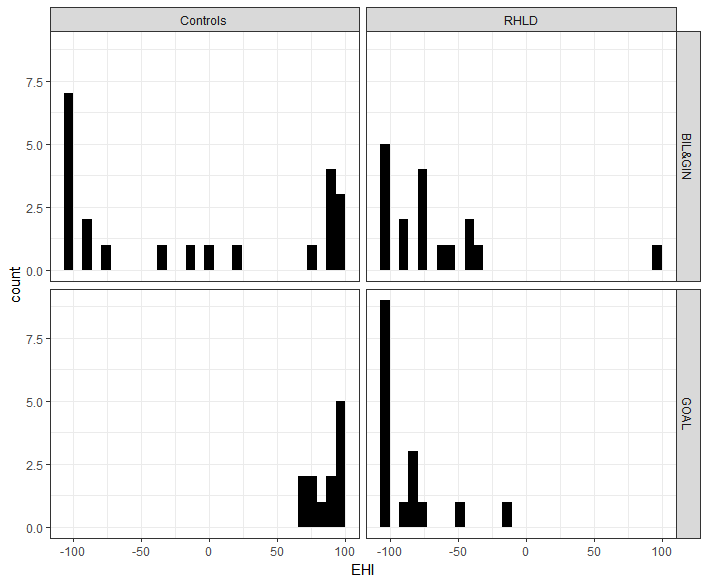


### Figure S2: Histogram distribution of the manual preference strength variable assessed by the Edinburgh Handedness Inventory (EHI) score across RHLD cases and controls, per dataset.


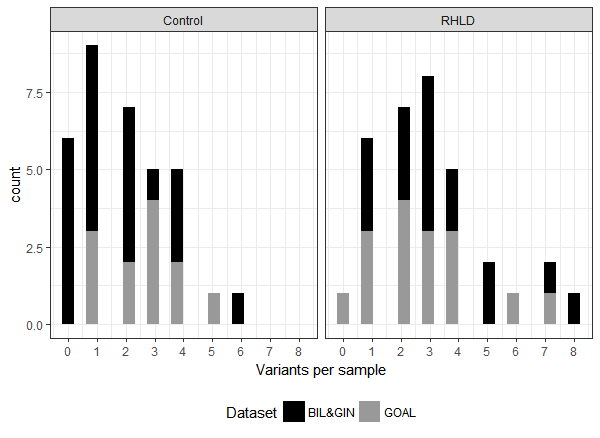


### Figure S3: Distribution of mutations within the ‘actin cytoskeleton’ gene set.
